# MUC5AC filaments illuminate the structural diversification of respiratory and intestinal mucins

**DOI:** 10.1073/pnas.2419717122

**Published:** 2025-03-04

**Authors:** Meital Haberman, Roman Kamyshinsky, Nava Reznik, Noa Yeshaya, Lev Khmelnitsky, Elizabeth G. Plender, Evan E. Eichler, Deborah Fass

**Affiliations:** ^a^Department of Chemical and Structural Biology, Weizmann Institute of Science, Rehovot 7610001, Israel; ^b^Department of Chemical Research Support, Weizmann Institute of Science, Rehovot 7610001, Israel; ^c^Department of Genome Sciences, University of Washington, School of Medicine, Seattle, WA 98195; ^d^Basic Sciences Division and Computational Biology Program, Fred Hutchinson Cancer Center, Seattle, WA 98109; ^e^HHMI, University of Washington, Seattle, WA 98195

**Keywords:** mucin, helical filaments, polymer, disulfide bonds, respiratory mucus

## Abstract

Secreted mucins are multimegadalton glycoprotein polymers that share the function of protecting mucosal tissues but diversified for activities in different organs of the body. Structural studies of secreted mucins are complicated by the enormous sizes, flexibility, and complex supramolecular assembly modes of these glycoproteins. The two major respiratory mucins are MUC5AC and MUC5B. Here, we present structures of a large amino-terminal segment of MUC5AC in the form of helical filaments. These filaments differ from filamentous and tubular structures observed previously for the intestinal mucin MUC2 and the partial mucin homolog VWF. Nevertheless, the MUC5AC helical filaments support the proposed mechanism, based on MUC2 and VWF, for how noncovalent interactions between mucin monomers guide disulfide crosslinking to form polymers. The high-resolution MUC5AC structures show how local and limited changes in amino acid sequence can profoundly affect higher-order assembly while preserving the overall folds and polymerization activity of mucin glycoproteins. Differences in supramolecular assembly are likely to be functionally significant considering the divergence of mechanical properties and physiological requirements between respiratory and intestinal mucins. Determining the high-resolution structures of respiratory mucins provides a foundation for understanding the mechanisms by which they clean and protect the lungs. Moreover, the MUC5AC structure enables visualization of the sites of human amino acid sequence variation and disease-associated mutations.

## Significance

Secreted respiratory “mucin” glycoproteins carry out routine cleaning of the lungs, respond to acute infections, and are involved in chronic diseases such as asthma and cystic fibrosis. Mucin supramolecular assembly and disulfide-mediated polymerization during biosynthesis provide these massive glycoproteins with the biophysical properties they need to serve as the first line of defense against inhaled pathogens. The high-resolution structure of a supramolecular assembly derived from a respiratory mucin shows how this mucin locks heads with itself to generate protective polymers.

Mucins and von Willebrand factor (VWF) are large glycoproteins that have different functions but share a disulfide-mediated polymerization mechanism (1). VWF polymers are secreted by platelets and endothelial cells and participate in hemostasis (2). Mucin polymers are secreted by goblet cells in epithelial tissues and protect and clean tissue surfaces (3, 4). Mucin and VWF polymerize in the late secretory pathway [i.e., downstream of the endoplasmic reticulum (ER)] (5), facilitated by low-pH-induced supramolecular assembly. Multiple glycoprotein dimers that were linked via their carboxy termini (“tails”) in the ER are thought to come together in the Golgi apparatus or secretory granules to form beaded filaments. At the center of each bead in the filament, reactive cysteines in the amino termini (“heads”) of separate dimers are juxtaposed, such that bonding of these cysteines yields “head-to-head, tail-to-tail” polymers (6).

Recombinant head segments of mucins and VWF recapitulate in vitro the process of supramolecular assembly that promotes polymerization (6). Evidence for this assertion comes from the study of VWF biosynthesis. In contrast to mammalian mucin storage granules, which are spherical structures with diameter ~10 µm, VWF storage granules, called Weibel-Palade bodies (WPBs), are smaller, cigar-shaped organelles with thickness of only a few hundred nanometers. These dimensions facilitated structural studies, using cryoelectron tomography, of naturally packaged, full-length VWF in WPBs (7). Helical reconstruction demonstrated that VWF self-assembles in cells as a tubule formed by interacting beads spiraling around a central axis (7). Importantly, the helical parameters of a coiled filament formed by a VWF amino-terminal segment in vitro (8, 9) matched the helical parameters of full-length VWF in WPBs (7), indicating that in vitro assembly of these glycoprotein fragments sheds light on physiologically relevant bioassembly processes. Though changing solution conditions or the span of the amino-terminal segment can modify the supramolecular assembly of recombinant mucins (10), the formation of beaded filaments is a recurrent feature in high-resolution studies of mucins to date and provides a mechanism for polymer formation (6). The current challenge is to determine whether and how higher-order interactions within or between beaded filaments during polymer production affect downstream mucus properties.

We present a study of the amino-terminal segment of MUC5AC, one of the two main respiratory mucins (11). MUC5AC is also expressed in the stomach (12) and by pancreatic, ovarian, and colon cancer cells (13, 14). Though normally produced at lower levels than MUC5B, which is the dominant mucin in healthy lungs, MUC5AC is induced upon infection (15), and high MUC5AC levels are protective against respiratory viruses (16). Differences in glycosylation between mucin paralogs have been detected (17), but little is known about how diversification has helped optimize mucins for routine functions and acute challenges. In this work, the high-resolution structures obtained for two helical filamentous forms of MUC5AC, determined by cryoelectron microscopy (cryo-EM), are described. These MUC5AC structures are then compared to the structures of filaments and tubules formed by a similar segment of the MUC2 intestinal mucin, a major secreted mucin that has also been analyzed structurally. This comparison shows how evolutionary divergence of mucin amino acid sequences, and the lengths of key natively disordered glycoprotein segments, affect mucin higher-order self-association.

## Results

### MUC5AC Beaded Filament Structure.

**Fig. 1.**
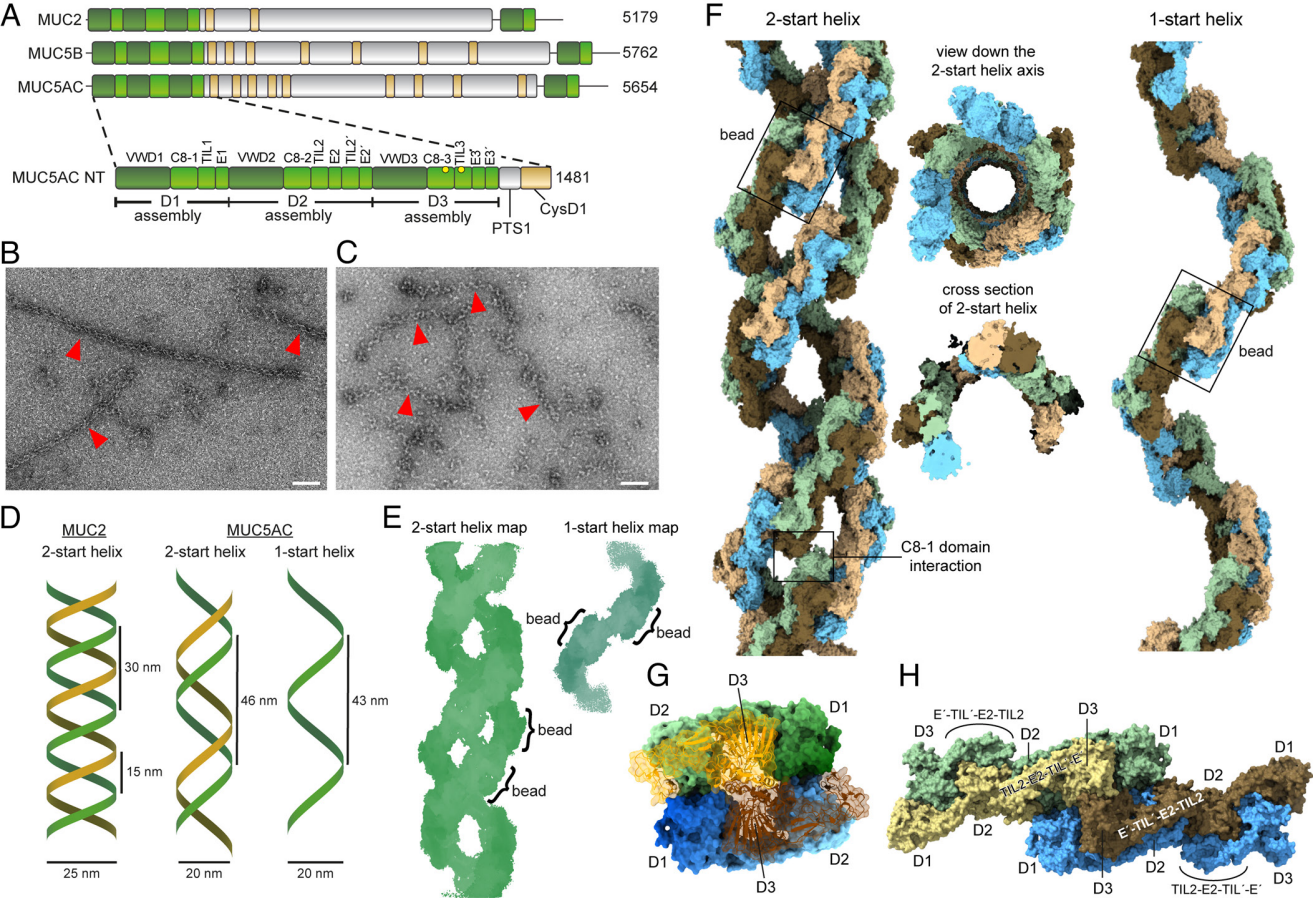
The amino-terminal segment of the MUC5AC mucin forms helical filaments in acidic solution corresponding to the pH of secretory granules. (*A*) Primary structure maps of the major secreted mucins. Folded regions are colored, and natively disordered regions rich in proline and glycosylated threonine and serine (PTS regions) are gray. Lengths in amino acids of the full-length protein and amino-terminal (NT) segment are shown to the right. TEM images showing fields rich in (*B*) two-start and (*C*) one-start MUC5AC NT helices, marked with red arrowheads. Scale bars are 100 nm. (*D*) Illustrations of MUC5AC NT helices compared to a helical supramolecular assembly previously observed for MUC2 (10). (*E*) Cryo-EM maps of MUC5AC NT helices. A smaller box size was used for reconstruction of the one-start helices. (*F*) Structure models of the two-start and one-start MUC5AC helices. Colors correspond to separate NT polypeptides. (*G*) Domain organization within a MUC5AC bead. Color gradients illustrate the antiparallel orientation of the D1 and D2 segments cradling the disulfide-bonded D3 domains. (*H*) Four polypeptides in the beaded filament illustrate how the TIL2-E2-TIL′-E′ segment links the D1 and D2 region in one bead to the D3 region of the same polypeptide in the adjacent bead.

The amino-terminal, head region of human MUC5AC (Fig. 1*A*) was produced in suspension-adapted HEK293 cells using similar methods as for a study of MUC2 (6). After incubation at acidic pH (*SI Appendix*, Fig. S1), the MUC5AC amino-terminal segment (hereafter MUC5AC) was observed by negative-stain transmission electron microscopy (TEM) to form helical structures (Fig. 1 *B* and *C*) that appeared to be one-start and, more commonly, two-start helices (Fig. 1*D*). Cryo-EM data were collected, and the two helical forms were analyzed separately (*SI Appendix*, Fig. S2). The 3D maps produced by single-particle reconstruction (Fig. 1*E*), treating helical segments as particles, revealed that the helices were composed of beaded filaments resembling those seen previously for MUC2 (6) and VWF (8, 9). In addition to the filament maps, a single-bead map was obtained at 2.9 Å resolution (*SI Appendix*, Fig. S3). An atomic coordinate model for a single bead was refined, and the resulting structure was docked into the two-start helix map and replicated using the helical parameters (Table 1) to provide a helix model.

**Table 1.** Mucin and von Willebrand factor (VWF) helical parameters

Protein	Axial rise per subunit (Å)	Helical twist	reference
MUC2 extended filament	134.7	98.1	Ref. 6
MUC2 helix	69.5	83.2	Ref. 10
VWF	27.6	83.1	Ref. 9
MUC5AC 1-start	89	75	This work
MUC5AC 2-start	94	74	This work

The MUC5AC helices (Fig. 1*F*) can be compared with VWF tubules (8, 9) and with a helical structure previously observed as an alternative supramolecular assembly state of the MUC2 beaded filament (10) (*SI Appendix*, Fig. S4). Unlike the MUC2 helix, which was two-start and exhibited D2 symmetry, the MUC5AC two-start helix lacks D2 symmetry. The intertwined MUC5AC filaments are related by a rotation around the helix axis of 114°, rather than 180°, and are offset from one another by a translation of about 22 Å along the helix axis. Modeling the insertion of a third filament using the parameters that relate the two observed filaments resulted in a steric clash with the subsequent helical turn, explaining why three-start helices were not observed. The helical parameters are similar for the one- and two-start helices, showing only a 5% deviation in the axial rise per subunit (Table 1 and *SI Appendix*, Fig. S5). The two filaments in the two-start helices interact with one another at their C8-1 domains, but neither helix type makes interactions between helical turns (Fig. 1*F*). Thus, the geometry of the MUC5AC helices is dictated primarily by the intrinsic angle between the successive beads in the helices. This arrangement differs from the VWF and MUC2 helices, which appeared to be stabilized by interactions between helical turns (8–10) (*SI Appendix*, Fig. S4).

**Fig. 2.**
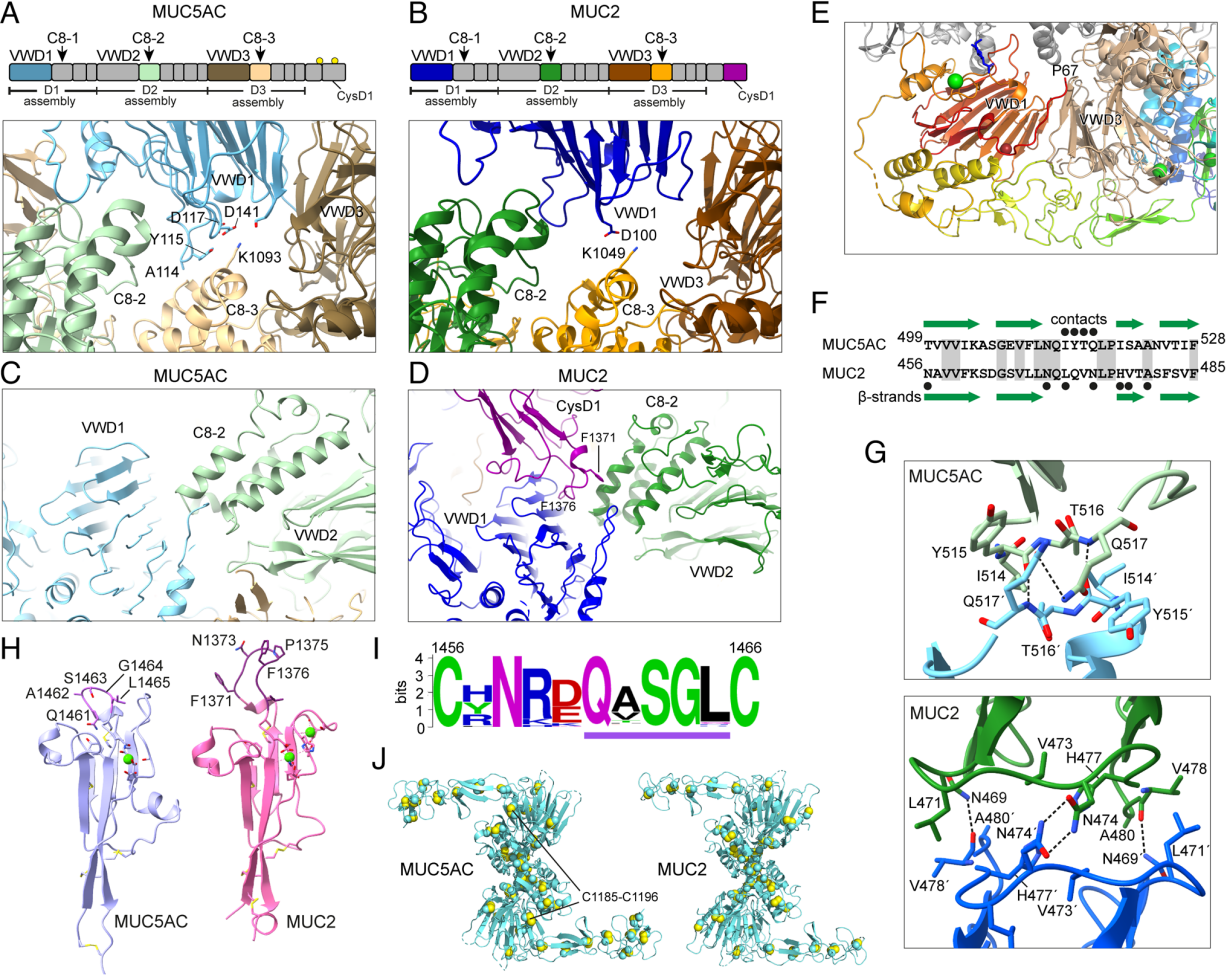
Interdomain interactions differ between MUC5AC and MUC2. (*A*) Packing of domains within the MUC5AC bead. The VWD1 domain interacts with the C8-3 domain at the center of the bead. Domains are color-coded according to the maps above the structure images, but it should be noted that the domains displayed in the structures do not belong to the same polypeptide but rather to each of the four distinct polypeptides that constitute a bead. (*B*) In the MUC2 bead, the VWD1 domain is more distant from the C8-3 domain, producing a slightly wider bead. (*C*) In MUC5AC, the VWD1 domain of one polypeptide in the bead is packed closely against the C8-2 domain of the symmetry-related polypeptide. (*D*) In MUC2, the CysD1 domain is wedged between the VWD1 and C8-2 domains. (*E*) The amino-terminal region upstream of the VWD1 domain interacts with an exposed β-strand in VWD3 of another polypeptide. P67 is the first amino acid visible in the cryo-EM map. (*F*) Sequence alignment of MUC5AC and MUC2 in the region of the VWD2 domain bead–bead interface. Green arrows are β-strands. Black circles indicate residues involved in intermolecular interactions. Amino acid identities are highlighted. (*G*) Structures of the VWD2 interbead interfaces. Primes indicate symmetry-related amino acids in the adjacent bead. Dashed lines show apparent hydrogen bonds. (*H*) AlphaFold 3 (18, 19) prediction of the MUC5AC CysD1 domain, which was not detected in the cryo-EM map of the MUC5AC NT, despite being present in the protein segment. The calcium ion (green sphere) was added at the equivalent position to one of the calcium ions observed in the MUC2 CysD1 structure, determined previously using X-ray crystallography (6). Amino acids in the principal bead-binding loop of MUC2 and the analogous, shorter loop of MUC5AC are labeled. Disulfide bonds and calcium-coordinating amino acid side chains are shown as sticks. (*I*) Sequence logo of the short loop in MUC5AC orthologs emphasizes the conservation of small amino acids (i.e., serine and glycine) at the tip of the loop. The amino acids shown in the loop in panel H are underlined in purple. (*J*) The D3 assemblies at the centers of the MUC5AC and MUC2 beads are very similar. A MUC5AC disulfide bond that does not have a counterpart in MUC2 is labeled.

The MUC5AC beads are similar in many respects to those seen previously for MUC2 and VWF but also show notable differences. Within each MUC5AC bead, two copies of the D1 and D2 segment, arranged antiparallel to one another, cradle the central pair of disulfide-bonded D3 assemblies (Fig. 1*G*), as in MUC2 and VWF. The two interacting D3 assemblies in MUC5AC are connected to the D1 and D2 segments from the two neighboring beads through interbead bridges composed of the TIL2/E2/TIL2′/E′ domains (Fig. 1*H*), as also seen for the related proteins (6, 8–10). A notable difference between MUC5AC and MUC2, however, involves the details of packing among domains within the beads. MUC5AC is more compact than MUC2, smaller by about 8 Å in both the long and the short axes of the oblong bead. The biggest difference leading to this compaction occurs at the D1 assembly. The MUC5AC VWD1 domain contacts both the VWD3 domain, located near the edge of the bead, and the C8-3 domain, located near the center of the bead (Fig. 2*A*). In contrast, the MUC2 VWD1 domain is set farther back from the bead center and contacts only the VWD3 domain, except for a salt bridge between D100 in VWD1 and K1049 in C8-3 (Fig. 2*B*). This difference between MUC5AC and MUC2 is due in part to the participation of the CysD1 domain in the MUC2 bead (Fig. 2 *C* and *D*) and will be described further below. A further interaction observed in MUC5AC was a segment of polypeptide upstream of the VWD1 domain participating in an intermolecular β-sheet by hydrogen bonding to an exposed β-strand in VWD3 (Fig. 2*E*).

Another major difference between MUC5AC and MUC2 is the rotation angle relating adjacent beads in the filaments (Table 1), accommodated by the flexibility or evolved differences in the interbead bridges and the bead–bead interface. While the three-dimensional structure of the VWD2 domain in the vicinity of the interface is conserved between MUC5AC and MUC2, amino acid sequence variation is seen in this region (Fig. 2*F*). The sequence differences accommodate a change in relative orientation of the VWD2 domains in two adjacent beads. The MUC2 VWD2 domains are positioned with their β-sandwiches in parallel at the bead–bead interface. In contrast, the juxtaposed MUC5AC VWD2 domain β-sandwiches are at an angle of about 45° to one another. In both filaments, the VWD2 domains are closely packed against one another, making both hydrophobic and hydrogen bonding interactions. In MUC5AC, these interactions involve I514, Y515, T516, Q517, and their symmetry-related residues (Fig. 2*G*). In MUC2, interbead interactions involve N469, L471, N474, H477, V478, A480, and perhaps N456 (Fig. 2*G*) (6). The D2 helical arrangement of MUC2 (10), in contrast, showed minimal VWD2–VWD2′ interactions, limited to a potential interaction of only L471 with its symmetry mate, suggesting that this supramolecular structure was stabilized instead by the interbead bridges and by interactions between helical turns.

### Role of the CysD1 Domain in the MUC5AC Beaded Filament.

In the study of MUC2, the helical arrangement of the amino-terminal region was observed only for a segment that lacked the CysD1 domain (10). In the presence of CysD1, MUC2 formed an elongated beaded filament stabilized by docking of two CysD1 domains onto the opposite side of each bead from the D3 assemblies (6). These CysD1 domains extended from the D3 assemblies of the two neighboring beads, facilitated by the intervening, natively disordered proline-, serine-, and threonine-rich, highly glycosylated PTS1 region (Fig. 1*A*). The MUC5AC amino-terminal segment studied in this work contained an analogous PTS1 region and the CysD1 domain. However, MUC5AC self-assembled into a helix rather than an elongated beaded filament, and cryo-EM map density was not detected for the CysD1 domain. Moreover, helical filaments were formed by the MUC5AC amino-terminal segment even after treatment with the StcE mucinase to cleave the PTS1 region and remove the CysD1 domain (*SI Appendix*, Fig. S6), indicating that a tethered CysD1 is not required for MUC5AC beaded filament formation. Comparing the structure of MUC5AC CysD1 predicted using AlphaFold 3 (18, 19) with the experimental structure of MUC2 CysD1 (6) (Fig. 2*H*), it is evident that MUC5AC CysD1 lacks the two exposed phenylalanines and proline by which MUC2 CysD1 interacted with the rest of the beaded filament (6). The comparable loop in MUC5AC is shorter and contains small amino acids (i.e., alanine, serine, and glycine) rather than aromatics (Fig. 2*H*). The length and sequence of this loop are highly conserved in MUC5AC from vertebrate orthologs (Fig. 2*I*), suggesting structural or functional constraints supporting its divergence from MUC2.

In contrast to the shorter loop seen in MUC5AC CysD1 compared to MUC2 CysD1, the PTS1 region of MUC5AC is longer than that of MUC2. Many mammalian MUC5AC PTS1 regions are between 62 and 68 amino acids in length, whereas MUC2 PTS1 segments typically range from 40 to 50 amino acids (6). Though demonstrated only for the human MUC2 ortholog, the latter range appears to be consistent with the length of the spacer needed to span between beads to dock the CysD1 domain onto the neighboring bead. Indeed, a path of weak map density corresponding to the PTS1 region was observed between each D3 assembly in one bead and a CysD1 domain attached to a neighboring bead of the extended MUC2 filaments (6). Not surprisingly, without an anchored CysD1 domain at the other end, the PTS1 region of MUC5AC was undetectable in the cryo-EM maps. TIL3 was the last domain that could be modeled from the MUC5AC cryo-EM density.

As noted above, a difference was detected between the MUC5AC and MUC2 bead structures in the packing of the VWD1 domain with the central D3 assemblies. This difference is due, at least in part, to the MUC2 CysD1 domain. The extended MUC2 CysD1 loop containing two phenylalanines (Fig. 2*H*) was seen wedged between VWD1 of one D1 and D2 segment and C8-2 of the antiparallel D1 and D2 segment in the bead (Fig. 2*D*). By distancing VWD1 from C8-2, the CysD1 wedge also distanced VWD1 from the C8-3 domain of the central, D3 assembly. Without a CysD1 domain present in the MUC5AC bead, the VWD1 domains are packed more tightly against C8-2, and consequently against C8-3 (Fig. 2*D*). Considering these differences in the positioning of the cradling domains in the bead, it is remarkable that the D3 assemblies, which form the intermolecular disulfide bonds that contribute to mucin polymerization, are almost identical in MUC2 and MUC5AC (Fig. 2*J*).

### Copper Binding by MUC5AC D1 Assembly.

**Fig. 3.**
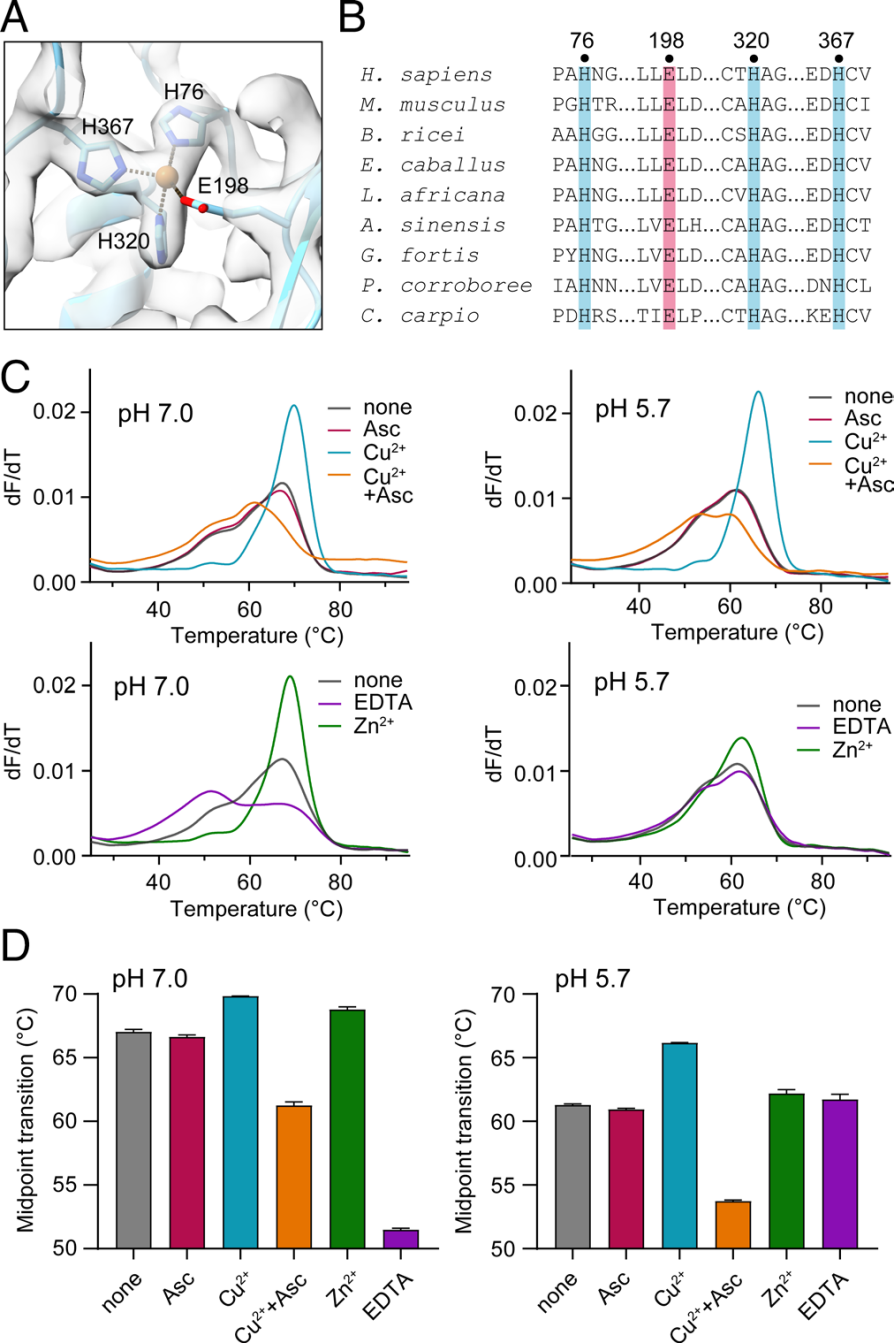
Copper binding by MUC5AC. (*A*) Metal binding site in the D1 assembly of MUC5AC helical filaments. Coordinating histidines and a nearby glutamic acid are labeled. Map density is contoured at 8σ. (*B*) Amino acid sequence alignment of MUC5AC orthologs highlighting the metal-binding residues. (*C*) Differential scanning fluorimetry (DSF) of MUC5AC D1. Ascorbic acid (Asc) reduces the added Cu^2+^ to Cu^1+^. Data are plotted as the first derivative of fluorescence as a function of temperature for experiments conducted in buffers at pH 7.0 and 5.7. (*D*) Plots of the temperature midpoints of the denaturation transitions. For samples with multiple transitions, the midpoint is reported for the highest peak in the dF/dT plot.

The structure of the MUC5AC bead enabled inspection of a copper-binding site anticipated from a study conducted on MUC2 and an amino acid sequence comparison among secreted mucin paralogs (20). Previous crystallographic, biochemical, and spectroscopic analyses revealed that the intestinal MUC2 is a specific copper-binding protein with distinct sites in the D1 assembly for Cu^2+^ and Cu^1+^ (20). Sequence alignments showed that human MUC5AC and MUC5B share with MUC2 the three histidines and one glutamic acid that compose the Cu^2+^ binding site, but the respiratory mucins lack the constellation of methionines that form the Cu^1+^ binding site (20). In the cryo-EM structures of MUC2 beaded filaments and helices (6, 10), the histidines and methionines were observed to point roughly toward one another without partitioning into the two distinct coordination environments for the two copper redox states detected in crystallographic and X-ray absorption studies of the isolated D1 assembly (20). The well-formed Cu^2+^ binding site seen in the Cu^2+^-bound MUC2 D1 crystal was disrupted in the beaded filament of the full amino-terminal segment by a rotation of the TIL1 domain away from its position in the crystal, displacing a key histidine (20). In the MUC5AC cryo-EM structures, side chain nitrogens of the three histidines in the putative Cu^2+^ binding site were seen at distances of about 2.1 Å from a central density (Fig. 3*A*), which appeared at 14σ in the bead map. Density for the glutamate carboxylate was ambiguous, but 6σ map density clearly showed the side chain oriented toward the histidines and their metal ligand. The histidines and glutamates are conserved in vertebrate MUC5AC orthologs (Fig. 3*B*).

The solution conditions that promoted robust filament formation of MUC5AC contained micromolar concentrations of zinc (see Material and Methods), but other metals may have been available from the cell expression system or purification buffers. The identity of the metal bound to the histidines in the MUC5AC cryo-EM structures could not be determined from the cryo-EM maps, so solution binding studies were performed on the isolated D1 assembly of MUC5AC to test for copper and zinc binding. Similarly to a previous observation for MUC2 D1 (20), the addition of Cu^2+^ shifted the thermal denaturation midpoint of MUC5AC D1 to higher temperatures (Fig. 3 *C* and *D*). In contrast, the lesser but still substantial thermal stabilization provided to MUC2 by Cu^1+^ [formed by reduction of Cu^2+^ using ascorbic acid (Asc)] did not occur for MUC5AC D1 (Fig. 3 *C* and *D*). In fact, Cu^1+^ was found to destabilize MUC5AC D1. Zn^2+^ stabilized MUC5AC D1 and increased the cooperativity of the thermal transition at pH 7.0 but had minimal effect at pH 5.7 (Fig. 3 *C* and *D*), as observed previously for MUC2 (20). Addition of EDTA at pH 7.0, but not at pH 5.7, shifted the major thermal transition to a lower temperature (Fig. 3 *C* and *D*), likely due to removal of calcium from a distinct, conserved site in mucins and VWF (21, 22).

### Sites of Genetic Diversity in MUC5AC.

**Fig. 4.**
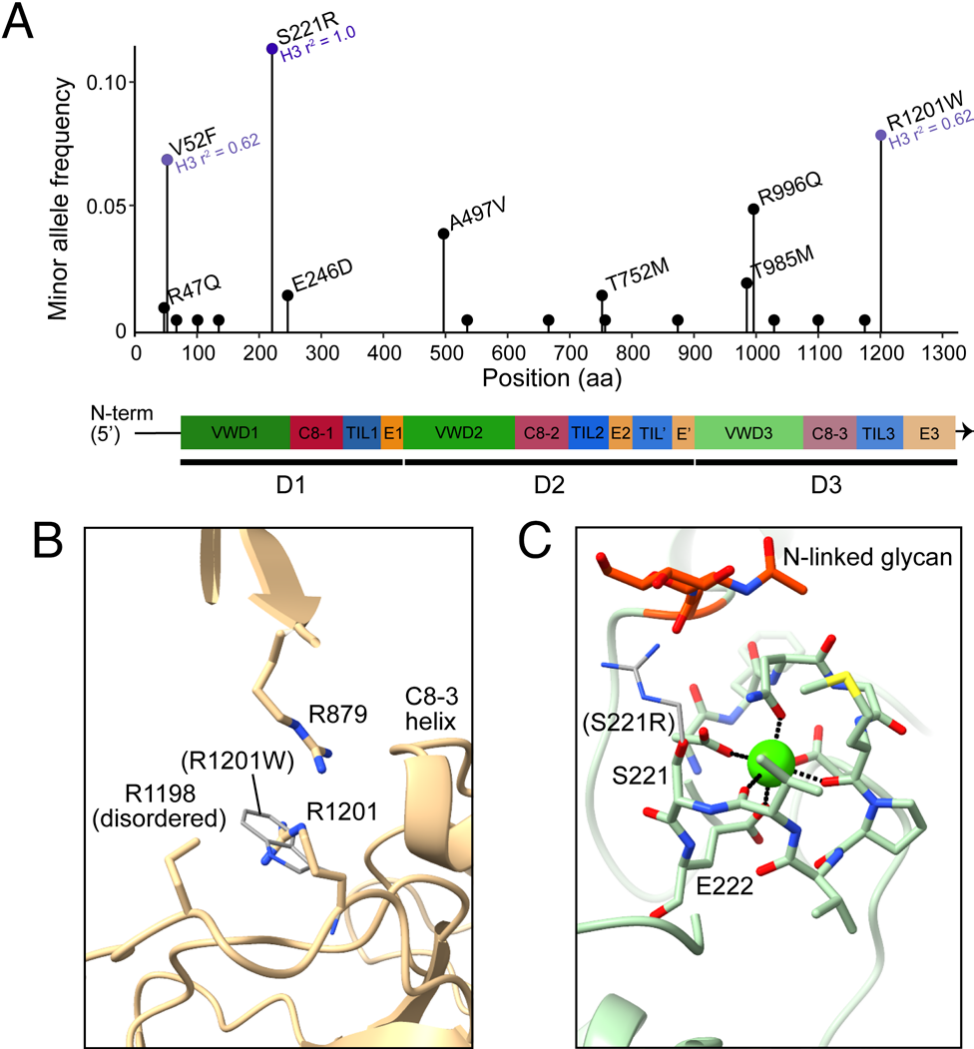
Variability in the amino-terminal region of human MUC5AC. (*A*) Nonsynonymous mutations in the N-terminal region of *MUC5AC* across 206 human haplotypes sequenced and assembled with long reads. Three mutations (V52F, S221R, and R1201W) feature minor allele frequencies >5%. H3 r^2^ values (purple text) correspond to squared correlations between the three common N-terminal variants and *MUC5AC* H3 alleles, as determined by Plender et al. (23). H3 alleles (MAF ~7%) feature elongated variants of *MUC5AC* (6,317 to 6,325 amino acids) with an additional PTS domain and CysD domains. (*B*) R1201 is found near other arginine residues near the carboxy terminus of a helix in the C8-3 domain. The R1201W variant (thin gray sticks) is modeled with a tryptophan rotamer that would not introduce steric clashes. (*C*) S221 is in a calcium-binding loop. The S221R variant (thin gray sticks) is modeled with an arginine rotamer that would place the side chain near an N-linked glycan.

MUC5AC shows substantial genetic diversity (23). The MUC5AC structure enabled analysis of the locations and roles of amino acid positions that exhibit polymorphism in the human population. We examined the segment of the MUC5AC gene encoding amino acid positions 1 through 1,230 and found 19 sites of variation. Most exhibit allele frequencies for the amino acid differences of less than 5%, with three exceptions (Fig. 4*A*). V52F is present in about 7% of haplotypes (n = 14), S221R is present in about 11% of haplotypes (n = 23), and R1201W is present in about 8% of haplotypes (n = 16). V52 is within a disordered segment of polypeptide upstream of the VWD1 domain that lacked corresponding density in the cryo-EM map, but S221 and R1201 are present within the MUC5AC structure model. R1201 is in the TIL3 domain. Though map density for the R1201 side chain is weak, and the residue may be sampling multiple rotamers, the side chain is evidently located near the side chains of R879 from the E′ domain and R1198, also from the TIL3 domain (Fig. 4*B*). Though charge repulsion would seem to disfavor such clusters of basic residues, arginine–arginine interactions are commonly found in protein structures (24). The MUC5AC arginine cluster is near the carboxyl end of a helix in the C8-3 domain and thus positioned to interact with the helix dipole. Substitution of a tryptophan for R1201 might enable a classical cation–π interaction to form with R879 or R1198 and may affect local structure or stabilization of the C8-3 helix. S221, in turn, is in the VWD1 domain. It is part of a calcium-binding loop and immediately precedes the calcium ligand E222 (Fig. 4*C*). The effect of mutation to arginine on calcium binding is difficult to determine from inspection of the structure, but an arginine side chain at this position would approach the glycan attached to N205. Using the structures presented in this work, it should be possible to further explore specific MUC5AC variants of interest.

## Discussion

Secreted mucin family members share the function of protecting exposed epithelial tissues from environmental hazards. However, different organ systems face different challenges, and these challenges change over time, such as during acute infection. Consequently, mucin paralogs have arisen and diversified to carry out specialized roles. In particular, the two respiratory mucins, MUC5B and MUC5AC, complement one another in protecting lung tissue. MUC5B is the primary mucin responsible for routine mucociliary clearance, whereas MUC5AC is induced during infection (15) and uniquely underlies airway hyperreactivity in allergy (25). Differences have been observed in the mesoscale morphologies and dynamics of secreted MUC5B and MUC5AC in cultured airway cells (26, 27) and animals (28, 29). Both respiratory mucins have diverged from MUC2, the intestinal mucin, which has evolved to be a dynamic, tunable material that houses and feeds the extensive commensal microbiota of the colon (30–32).

A notable difference between the respiratory and intestinal mucins is the number of CysD domains and intervening PTS segments. In humans, MUC5AC and MUC5B typically have nine and seven CysD domains, respectively, whereas MUC2 has only two (Fig. 1*A*). The first CysD domain (CysD1) of human MUC2 was observed to participate in the self-assembly of linear beaded filaments by the amino-terminal segment of the glycoprotein under Golgi-mimicking conditions (6). In contrast, the CysD1 domain of human MUC5AC was not detected in association with the helical beaded filaments described here. Inspection of the Alphafold 3 model (18, 19) for the MUC5AC CysD1 domain, likely to be reliable due to the conservation of the five disulfide bonds in this tightly cross-linked fold family, reveals major differences from the X-ray crystal structure of the MUC2 CysD1 domain in the loop constrained by a disulfide bond between C1365 and C1379 (MUC2 amino acid numbering). This CysD1 loop made extensive interactions with the bead in the cryo-EM structure of MUC2 filaments (Fig. 2*D*) (6). The comparable loop in MUC5AC CysD1 is four amino acids shorter and lacks the two exposed phenylalanines and a proline that make interdomain interactions in MUC2 (Fig. 2*H*). These observations show that the reutilization of the CysD fold within and between mucin paralogs provides a stable scaffold upon which new interaction capabilities can potentially evolve.

A few possible explanations can be considered for the lack of a MUC5AC CysD1 domain interaction in the helical filaments described herein. The solution conditions used for beaded filament assembly may have been incompatible with CysD1 binding. Another possibility is that the MUC5AC CysD1 domain may naturally interact with MUC5AC segments outside the large amino-terminal fragment used for the cryo-EM studies. Alternatively, MUC5AC CysD1 may interact with other molecules, such as, hypothetically, MUC5B. Though the MUC2 CysD1 domain participated in assembly of beaded filaments at Golgi pH, CysD domains may make other interactions at other stages of mucin bioassembly or function. For example, CysD domains may become cross-linked by isopeptide bonds after secretion (33). Differences in CysDs (34) and their interactions are starting to be revealed, but the full functional diversity of these domains largely remains to be explored. The MUC5AC structure establishes that different mucin supramolecular assemblies are stabilized in different ways: MUC2 uses the CysD1 domain to promote beaded filament formation (6), whereas MUC5AC uses tighter packing between domains (Fig. 2*A*) and the participation of an N-terminal flexible segment (Fig. 2*E*) to strengthen multimolecular associations.

Previous work showed that recombinant mucin segments can assume very different supramolecular assembly states under slightly different solution conditions with the addition or subtraction of a single domain. Specifically, linear filaments and helical tubules were observed for MUC2 amino-terminal fragments containing or lacking the CysD1 domain, respectively (6, 10). This observation raises the question of whether the helical tubules reported here for MUC5AC are an artifact of studying a truncated fragment of the full-length protein. As noted in the Introduction, study of the mucin-related protein VWF demonstrated the physiological relevance of beaded filaments for these disulfide-bonded polymeric proteins (7). Nevertheless, care must be taken regarding the details of the helical assemblies, as it was previously shown that a VWF fragment lacking the A1 domain formed a tubule with slightly different helical parameters than those of the native protein (9, 10). While not dismissing this potential caveat, we propose that many of the differences seen between MUC5AC and MUC2 represent evolutionary divergence rather than accidental effects of experimental conditions. Notably, the internal domain organization within the MUC2 bead was identical in the linear and helical assembly states (6, 10). Though the MUC2 helical tubules lacked the CysD1 wedged between the VWD1 and C8-2 domains, the beads in the tubule retained the same relative orientations and interactions among remaining domains even in the absence of CysD1. The striking difference in appearance of the two MUC2 supramolecular assemblies, linear *vs*. tubular, was due merely to a change in packing angle and interface between beads, facilitated by local flexibility in the interdomain bridge near the TIL’ domain. In the case of MUC5AC, the differences in domain interactions seen in comparison to MUC2 are distributed throughout the interior of the bead and appear to be accommodated by many amino acid differences at the divergent interfaces.

In combination, the differences seen between MUC2 and MUC5AC exhibit a molecular logic. According to sequence and structural homology with VWF, we hypothesized that mucin beaded filaments represent in vivo bioassembly intermediates for polymerization (6). Extending the comparison, we expect the middle and carboxy-terminal portions of full-length MUC5AC to be arrayed around the beaded filaments analogous to how the VWF A and C domains project outward from the central tubule in WPBs (35). In the MUC5AC helices, the face of the bead corresponding to the docking site for CysD1 in the MUC2 beaded filaments (6) points inward, toward the helix axis. If the MUC5AC CysD1 domain were to pack against this back face of the bead, it would be sterically challenging for the rest of the protein to project outward, away from the helix. Thus, a lack of CysD1 binding to the helical filament solves this potential problem.

Diversification of supramolecular assembly modes is accompanied by diversification of a metal-binding site in mucins. MUC2 was previously shown to bind copper in two distinct oxidation states at two distinct but juxtaposed sites in the protein (20). Amino acid sequence alignments suggested that the respiratory mucins maintain the Cu^2+^ binding site but lack the ability to bind Cu^1+^, a prediction supported by the thermal denaturation experiments presented herein (Fig. 3 *C* and *D*). Though both Cu^2+^ and Zn^2+^ increased the denaturation temperatures of MUC5AC D1 and MUC2 D1 at neutral pH, further binding and crystallographic studies of MUC2 D1 demonstrated a marked preference for Cu^2+^ (20). A thorough investigation of the metal-binding specificity of MUC5AC will be required for a deeper understanding of the similarities and differences between lung and intestinal mucins. Remarkably, however, the current study revealed that supramolecular assembly can have different effects on metal binding in different mucins, a phenomenon that is difficult to predict from amino acid sequence. In the MUC5AC helical filament, the histidines were appropriately positioned to coordinate metal (Fig. 3*A*), whereas the MUC2 filaments and tubules reported previously (6, 10) showed distorted copper-binding sites and appeared to be metal-free (20). If the beaded filament and helical mucin structures indeed represent intracellular polymerization and packaging intermediates prior to secretion (6), the ability or inability to bind copper in these intermediates may provide hints regarding the physiological role of copper binding by these complex glycoproteins.

In addition to evolutionary diversification from one another, mucins show extensive diversity at their respective genetic loci (36). MUC5AC harbors structural polymorphisms that include variation in PTS domain copy number, CysD domain copy number, and tandem repeat motif usage (23, 37). At the genetic level, MUC5AC alleles can be classified into three common haplogroups of variants that range substantially in total predicted protein size (H1 = ~5,654 amino acids, H2 = ~5,742 amino acids, and H3 = ~6,325 amino acids) (23). In the N-terminal region of MUC5AC, we found that three nonsynonymous common variants (V52F, S221R, and R1201W) are disproportionately found (r2 = 0.62 to 1.00) on the same haplotypes as H3 variants, indicating that functional variation in these regions must be considered within the context of an elongated MUC5AC protein. In fact, S221R (rs35783651) is a tagging SNP for H3 variants (23) and is an expression quantitative trait locus (eQTL) for decreased expression of MUC5AC in pediatric asthma (38). R1201W (rs878913005) has been positively associated with Chronic Obstructive Airway Disease (COPD) and Idiopathic Pulmonary Fibrosis (39). However, it remains unclear whether the causative agent of this association is the variant itself or the background H3 haplotype.

The study of mucin molecular mechanisms and disease associations is complicated by the enormous sizes of these glycoproteins and their complex conformational variability and self-association properties. Nevertheless, cryo-EM has enabled elucidation of structural similarities and differences between mucin paralogs in a state hypothesized to represent a presecretion, compact form, providing insight into the diversification of these glycoproteins during evolution. Understanding mucin diversification will in turn provide insight into the selective pressures on their functions in various biological niches. The findings described here reveal that the amino-terminal region of the respiratory mucin MUC5AC assembles into loose helical tubules that do not display a docking site for the CysD1 domain, suggesting that this domain is free to make other interactions during MUC5AC self-assembly in low pH compartments. The structures of MUC5AC helical filaments also strengthen the generality of the bead-based mechanism for bringing D3 assemblies together for disulfide bonding and polymer formation (6, 8). With this generality established, it is now possible to investigate how variation in the coiling and interactions of mucin beaded filaments on the scale of tens or hundreds of nanometers may predispose these glycoproteins to the morphological diversity observed for secreted mucin strands and networks on the scale of tens and hundreds of microns (26–29).

## Materials and Methods

### Protein Production and Purification.

The plasmid for producing the amino-terminal segment of MUC5AC (human MUC5AC residues 28 to 1483) was based on pcDNA3.1. Coding sequence for the signal peptide of the protein QSOX1, a His_6_ tag, and a tobacco etch virus (TEV) protease cleavage site (MRRCNSGSGPPPSLLLLLLWLLAVPGANAAPQGHHHHHHENLYFQG) was fused upstream of a synthetic gene encoding the designated segment of MUC5AC. The plasmid was propagated in and purified from cells of the *E. coli* XL-1 strain. Proteins were produced by transient transfection of plasmid into suspended HEK293F cells grown in FreeStyle™ 293 Medium (ThermoFisher). Cells were split to 0.7 million cells per milliliter one day before transfection. Transfection was performed using the PEI Max reagent (Polysciences, Inc.) with a 1:3 ratio (w/w) of DNA to PEI. Six days after transfection, the culture medium was collected and centrifuged for 10 min at 500 g to pellet cells. The supernatant was then centrifuged for 10 min at 9,200 g to pellet any remaining particulate matter. The supernatant from this second centrifugation was filtered through a 0.45 μm filter, and the His_6_-tagged proteins were purified by nickel-nitrilotriacetic acid (Ni-NTA) chromatography. The buffer was then exchanged to 10 mM Tris, pH 7.5, 50 mM NaCl, and the proteins concentrated to 10 mg/ml using a centrifugal concentrator with 50 kDa cut-off. Protein was stored at 4 °C and used within a few days of purification.

The plasmid for producing the MUC5AC D1 assembly encoded residues 28 to 431 downstream of the QSOX1 signal peptide, His_6_ tag, and TEV cleavage site. The D1 assembly was produced as for the larger amino-terminal segment, but the protein was subjected to TEV cleavage during dialysis against phosphate-buffered saline following the Ni-NTA chromatography. The cleaved tag and the TEV protease were then removed using Ni-NTA beads. The buffer was exchanged to 10 mM MOPS pH 7.0, 100 mM NaCl, and 10% glycerol, and the protein was flash-frozen and stored at −80 °C until use.

The StcE expression plasmid was constructed in the laboratory of Natalie Strynadka (40) and obtained from the laboratory of Carolyn Bertozzi (41). The protein was purified essentially as described (41).

### Sample Preparation for EM.

For negative staining, the purified MUC5AC amino-terminal segment was diluted to a concentration of 0.45 mg/ml in 100 mM MES, pH 5.2, 45 µM ZnCl_2_, 1 M NaCl, and 10 mM CaCl_2_ and incubated at 37 °C overnight (~15 h). The pH scan (*SI Appendix*, Fig. S1) was conducted in the same solution except that the pH of the MES was altered. For samples treated with StcE protease, 11 mg/ml MUC5AC in 10 mM Tris, pH 7.5, 50 mM NaCl was left overnight at room temperature in the presence of 8, 4, or 2 µM StcE. Cleavage was verified by gel electrophoresis (*SI Appendix*, Fig. S6). The sample containing 2 µM StcE was then diluted to 0.45 mg/ml MUC5AC in 100 mM MES, pH 5.2, 45 µM ZnCl_2_, 1 M NaCl, and 10 mM CaCl_2_ and incubated at 37 °C overnight. After incubation, 3 µl of the protein solutions were applied to glow-discharged carbon-coated 300 mesh copper grids (Electron Microscopy Sciences) for 45 s, followed by staining with 2% uranyl acetate solution. Samples were visualized using a Tecnai T12 Spirit transmission electron microscope (Thermo Fisher Scientific) equipped with a OneView camera (Gatan).

For cryo-EM, purified MUC5AC amino-terminal segment was incubated at a concentration of 0.45 mg/ml in 100 mM MES, pH 5.2, 45 µM ZnCl_2_, 1 M NaCl, and 10 mM CaCl_2_ overnight (~15 h). Three µl of the protein solutions were applied to glow-discharged (Pelco easiGlow, Ted Pella) UltrAuFoil R 1.2/1.3 300 mesh gold grids (Electron Microscopy Sciences). Grids were plunge-frozen into liquid ethane cooled by liquid nitrogen using a Vitrobot Mark IV plunger (Thermo Fisher Scientific) at 100% humidity, 22 °C, −1 blot force, 3.5 s blot time.

### Cryo-EM Image Acquisition.

Cryo-EM data were collected on a Titan Krios G3i transmission electron microscope (Thermo Fisher Scientific) operated at 300 kV. Movies were recorded on a K3 direct detector (Gatan) installed behind a BioQuantum energy filter (Gatan) using a slit of 20 eV. Movies were recorded in counting mode at a nominal magnification of 105,000×, corresponding to a physical pixel size of 0.8242 Å. The dose rate was set to 17.6 e^−^/pixel/s, and the total exposure time was 1.6 s, resulting in an accumulated dose of 45 e^−^/Å^2^. Each movie was split into 40 frames of 0.04 s. Nominal defocus range was −0.6 to −1.5 μm. The microscope was optically aligned for fringe-free illumination, enabling reduction of the beam diameter to 710 nm. A total of 32,278 micrographs were collected in aberration-free image shift (AFIS) mode in EPU 3.6 (Thermo Fisher Scientific).

### Cryo-EM Image Processing.

Image processing was performed using CryoSPARC software (v4.4.1 to v4.6.0). The processing scheme is outlined in *SI Appendix*, Fig. S2. After being subjected to patch motion correction and patch CTF estimation, 22,193 movies were selected for further processing.

Particles used downstream for the one-start helix map were initially picked on a subset of micrographs using Blob Picker. Extracted particles (800-pixel box size Fourier-cropped to 64-pixel box size) were iteratively 2D classified, and their class averages were used as templates for the Template Picker job. After multiple rounds of 2D classification, selected class averages resembling one-start helices were used as templates for automated particle picking with Filament Tracer utility on all micrographs. After several rounds of 2D classification, 130,956 particles were extracted with an 800-pixel box size and carried forward for an ab initio reconstruction. Homogenous refinement with C2 symmetry imposed produced a 3.58 Å resolution map. A mask around a single bead was constructed using Segger (42). Local refinement with this mask improved the resolution to 3.38 Å with the goal of comparing the bead–bead interface (included in the map) to that of the two-start helix.

A higher-resolution map of a single bead was obtained by focusing on two-start helices. Initial particle picking was performed using Filament Tracer without templates. 124,295 particles were extracted with a box size of 700 pixels Fourier-cropped to 350 pixels, and 2D classified. Class averages resembling two-start helices were used as templates for Filament Tracer, followed by several rounds of 2D classification. A total of 198,256 particles extracted with a 550-pixel box size were used for ab initio reconstruction, followed by nonuniform 3D refinement resulting in a 3.33 Å resolution map. The obtained helical parameters were used for downstream helical refinement. Local refinement with a mask around a single bead yielded a 2.91 Å resolution map.

To generate a map of a full turn of the two-start helix, the same pool of particles was re-extracted with a 900-pixel box size Fourier-cropped to 448-pixels. A helical refinement with 165,735 particles was done with an ab initio initial model. The refinement produced a 3.90 Å resolution map.

### Model Building, Refinement, and Analysis.

A model of MUC5AC residues 28 to 1,320 was generated using AlphaFold2 colab (18). Domains of the model were fit into the single-bead cryo-EM map with the aid of Coot (43), then manually rebuilt. Refinement was performed using Phenix (44). The final structure models were analyzed for quality using Molprobity (45), scoring 100th percentile in both overall Molprobity and clashscore for the relevant resolution range (2.91 ± 0.25 Å) with no Ramachandran or bond/angle outliers. The EMRinger (46) score was 3.6. Structure images were prepared using ChimeraX v1.3 (47). Helical parameters were calculated using the draw_rotation_axis.py script and the draw_axis command in PyMOL (48).

### Differential Scanning Fluorimetry (DSF).

Protein stocks of 32 μM MUC5AC D1 in 10 mM MOPS pH 7.0, 100 mM NaCl, and 10% glycerol were diluted to a final concentration of 10 μM in 100 mM MOPS pH 5.7 or pH 7.0, 100 mM NaCl, and 0.05% Tween. The final glycerol concentration was 3.1% after dilution. Copper or zinc was added to a final concentration of 10 μM (1:1 molar ratio) and incubated for 15 min at room temperature with the protein. Asc was added, when relevant, to a final concentration of 200 μM before the measurement. Measurements were carried out in quadruplicate using a nano-DSF Prometheus Panta instrument. The samples were heated from 25 to 95 °C at a rate of 1 °C per minute. Excitation was at 280 nm, and the fluorescence emission ratio 350/330 nm was recorded. The melting temperature was identified as the point with the steepest slope in a plot of emission ratio as a function of temperature.

### Genetic Analyses for Common N-Rerminal Nonsynonymous Mutation.

MUC5AC haplotypes (n = 206) from long-read genome assemblies in ref. 23 were analyzed for common N-terminal nonsynonymous mutations. The three common variants (V52F, S221R, and R1201W) were analyzed for correlation (r2) with the three common haplogroups of MUC5AC alleles (H1, H2, and H3) using PLINK v1.9.

## Supplementary Material

Appendix 01 (PDF)

Movie S1. Animation of MUC5AC two-start helix map and model.

## Data Availability

The atomic coordinates data have been deposited in the Protein Data Bank with the codes 9GVJ (bead) (49) and 9GVQ (two-start helix) (50). Cryo-EM density maps have been deposited in the Electron Microscopy Data Bank with accession codes EMD-51636 (bead) (51) and EMD-51639 (2-start helix) (52).
