# Supplementary material for: MUC5AC filaments illuminate the structural diversification of respiratory and intestinal mucins
Source: Proc Natl Acad Sci U S A. 2025 Mar 4;122(10):e2419717122. doi: 10.1073/pnas.2419717122 (PMC11912381; doi:10.1073/pnas.2419717122)
Supplement: Supplementary file 1 — Appendix 01 (PDF) [file pnas.2419717122.sapp.pdf]

PNAS

Supporting Information for

MUC5AC Filaments Illuminate the Structural Diversification of Respiratory and Intestinal Mucins

Meital Haberman[1], Roman Kamyshinsky[2], Nava Reznik[1], Noa Yeshaya[1], Lev Khmelnitsky[1], Elizabeth G. Plender[3,4], Evan E. Eichler[3,5], and Deborah Fass[1*]

[1]Department of Chemical and Structural Biology, Weizmann Institute of Science, Rehovot, 7610001, Israel

[2]Department of Chemical Research Support, Weizmann Institute of Science, Rehovot, 7610001, Israel

[3]Department of Genome Sciences, University of Washington, School of Medicine, Seattle, WA 98195, USA

[4]Basic Sciences Division and Computational Biology Program, Fred Hutchinson Cancer Center, Seattle, WA 98109, USA

[5]Howard Hughes Medical Institute, University of Washington, Seattle, WA 98195, USA

Email: deborah.fass@weizmann.ac.il

This PDF file includes:

Figures S1 to S6

Other supporting materials for this manuscript include the following:

Movie S1

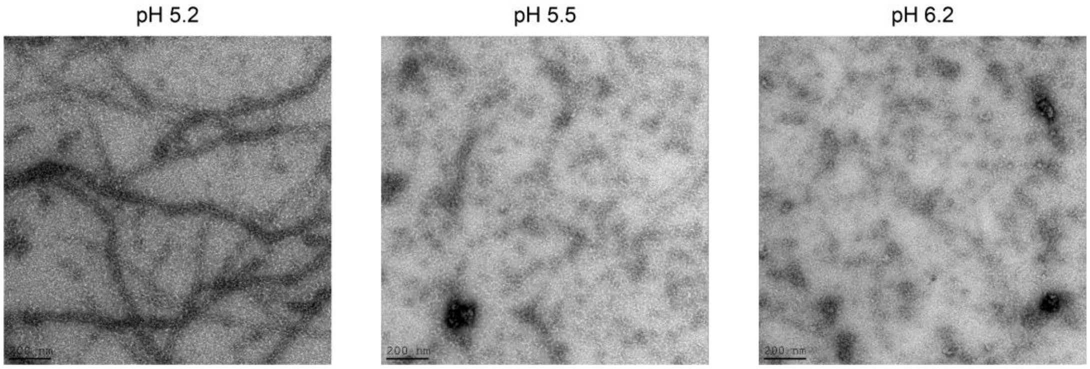

Fig. S1. MUC5AC helical filament formation as a function of pH. The amino-terminal segment of MUC5AC was incubated overnight in solutions containing 100 mM MES at the indicated pH values, 45 µM $ZnCl_2$, 1 M NaCl, and 10 mM $CaCl_2$. Self-assembly into helical filaments was observed in the range of pH 5.2 to 6.2, but filamentation was most robust at pH 5.2, as detected by negative-stain transmission electron microscopy. Scale bars are 200 nm.

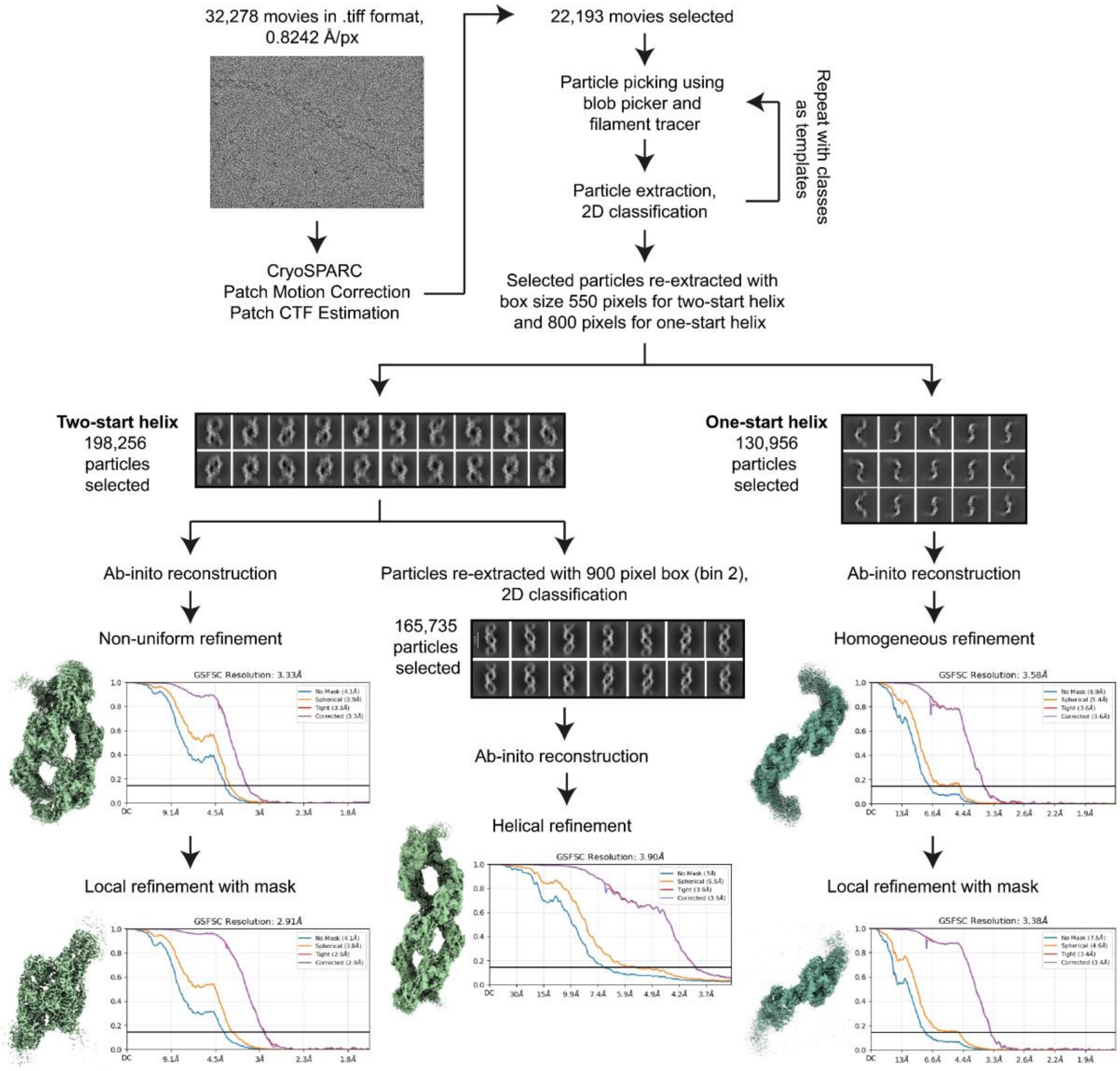

Fig. S2. Graphical summary of the cryo-EM data processing workflow for MUC5AC helical beaded filaments. Fourier shell correlation curves are displayed next to the associated maps. Resolution at the gold standard Fourier shell correlation (GSFSC) cut-off (0.143) is shown above each plot.

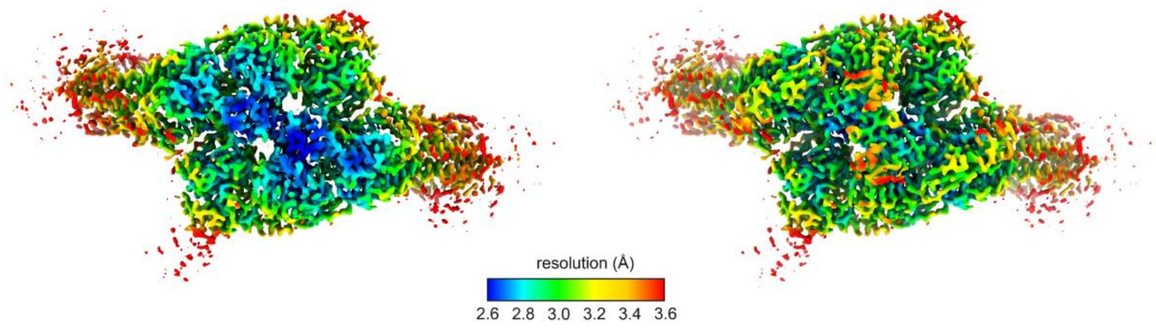

Fig. S3. Cryo-EM map of a single bead from the MUC5AC two-start helix, colored according to local resolution estimate. The right image shows the surface of the bead. The left image shows a cutaway view through the center of the bead.

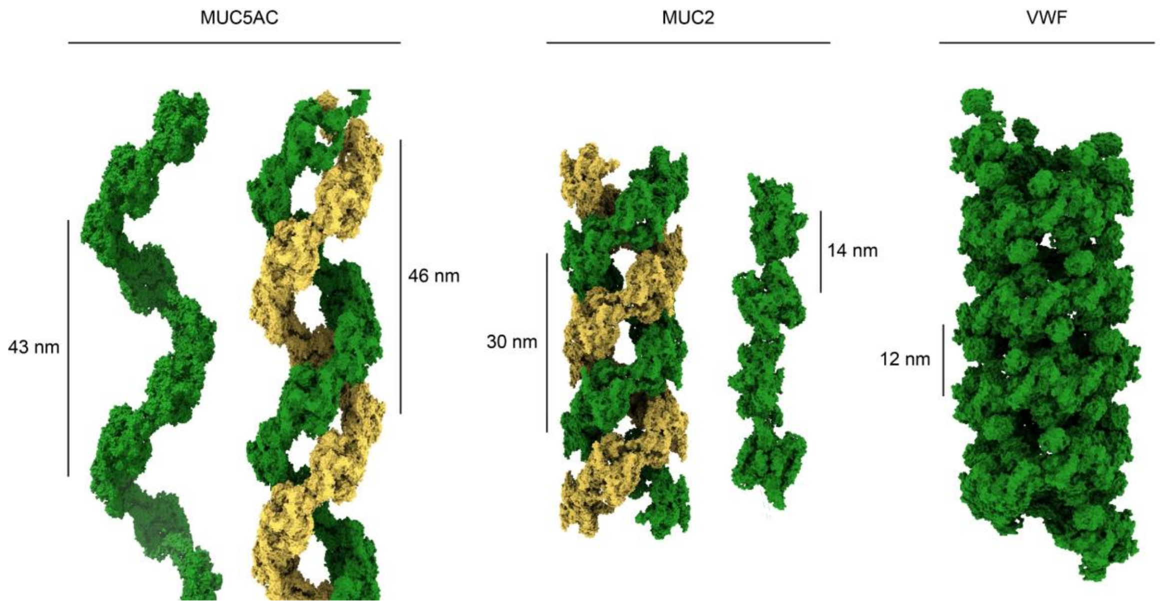

Fig. S4. Comparison of MUC5AC beaded filaments with filaments and tubules of MUC2 and VWF. MUC5AC filaments are reported in this work and are derived from PDB code 9GVJ subjected to the helical symmetry of the one-start and two-start helices. The MUC2 helical tubule was generated from PDB code 7PP6. The MUC2 elongated filament was generated from PDB code 6TM2. The VWF tubule was generated from PDB code 7ZWH. Two-start helices are colored green and yellow. The pitches of the helices, or the distance between bead centers for the MUC2 elongated filament, are indicated.

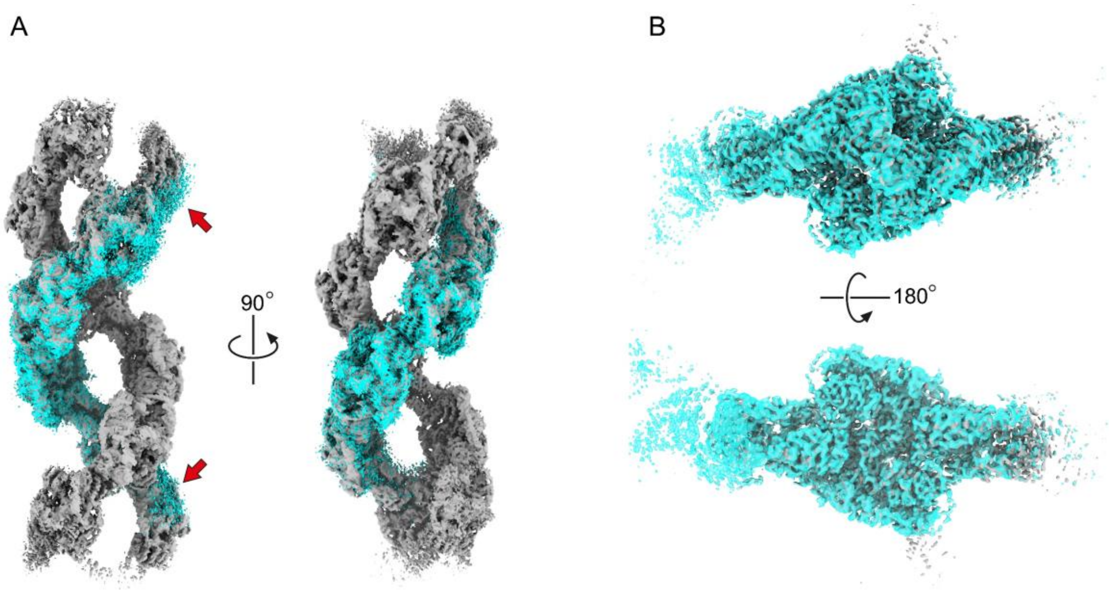

Fig. S5. Comparison of beads in the two-start and one-start MUC5AC helices. (A) Cryo-EM map of the MUC5AC one-start helix (cyan) superposed on the map of the two-start helix (gray). The helical parameters are similar, but the slightly shorter axial rise per subunit of the one-start helix can be seen by the poor superposition in the regions indicated by the red arrows. (B) Superposed masked maps covering one bead of the one-start (cyan) and two-start (gray) helices emphasize the overall structural similarity of the beads in the two helical forms.

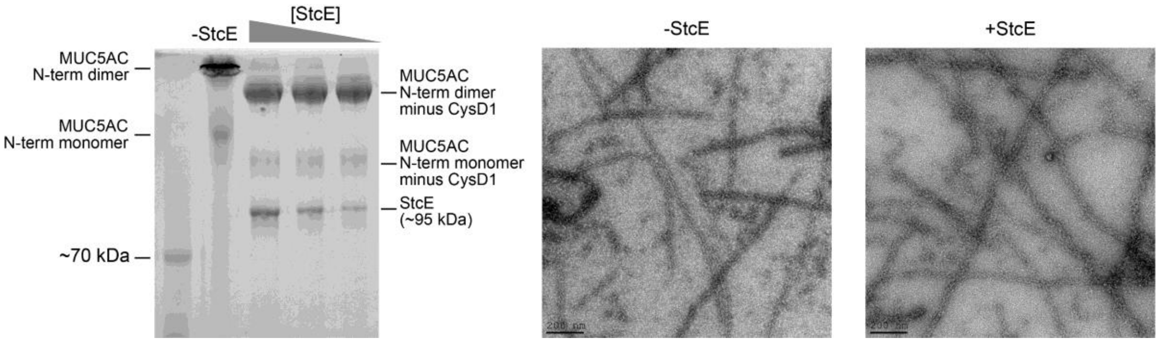

Fig. S6. Removal of CysD1 from the amino-terminal segment of MUC5AC does not prevent helical filament formation. On the left is a 7.5% denaturing polyacrylamide gel showing the intact purified MUCAC amino-terminal (N-term) segment. This species migrates very slowly through the separating gel due to its large size and extensive O-linked glycosylation. Cleavage of the MUC5AC amino-terminal segment with the StcE mucinase removes the CysD1 domain due to cleavage in the PTS1 region. On the right are negative-stain transmission electron micrographs of filaments formed without (-StcE) and with (+StcE) treatment, which demonstrate that a tethered CysD1 domain is not required for MUC5AC filament formation, as expected from the lack of an observed docking site for this domain in the helical filaments. Scale bars are 200 nm.

Movie S1 (separate file). Animation of MUC5AC two-start helix map and model.
